# Survival Improvement in Patients with Renal Cell Carcinoma and Disparities between Different Sexes, Races, and Socioeconomic Status: 1977–2016

**DOI:** 10.1155/2022/1587365

**Published:** 2022-07-30

**Authors:** Dijun Ouyang, Huanhuan Sun, Nan Chen, Yan Yan, Haiqing Ma, Jianchuan Xia

**Affiliations:** ^1^Department of Experimental Research, State Key Laboratory of Oncology in South China, Collaborative Innovation Center for Cancer Medicine, Sun Yat-sen University Cancer Center, Guangzhou 510060, Guangdong, China; ^2^Department of Oncology, The Fifth Affiliated Hospital of Sun Yat-sen University, Zhuhai 519000, Guangdong, China; ^3^Medical Research Center, Guangdong Provincial People's Hospital, Guangdong Academy of Medical Sciences, Guangzhou 510060, Guangdong, China; ^4^Department of Oncology, Guangdong Provincial People's Hospital, Guangdong Academy of Medical Sciences, Guangzhou 510060, Guangdong, China

## Abstract

**Objective:**

Rare research of renal cell carcinoma (RCC) has been made in a comprehensive and full description based on a long period of time as yet. This study was aimed at investigating the incidence and relative survival rates (RSRs) of RCC in the past forty years and to disclose the impact of sex, race, and socioeconomic status (SES) on RCC.

**Methods:**

The data as variables, including age, gender, race, and SES, were obtained from the Surveillance, Epidemiology, and End Results (SEER) database. SES was divided into three levels: low poverty, medium poverty, and high poverty. The medium- and high-poverty groups were integrated into one group in all analyses. The RSRs were calculated using period analysis methodology. Summary statistics including incidence and RSRs were analyzed by Kaplan–Meier and Cox proportional hazards models with GraphPad Prism 8.0.1 software and Stata 12.0 software.

**Results:**

A total of 77,513 patients diagnosed with RCC were enrolled in this study, showing an increased incidence and 10-year RSRs from 1977 to 2016. Patients older than 60 years had the highest incidence and the lowest RSRs. This research also showed significant disparities between different groups: incidence in males, blacks, and medium-high poverty groups was higher than that in females, whites, and low poverty groups, while RSRs were lower. For sex groups, the disparity of RSRs was obvious among patients who were 30–59 years old, but not among those younger than 29 years or older than 60 years. Based on SES, the survival gaps between different SES groups were getting wider over the past forty years.

**Conclusion:**

This study showed how age, sex, race, and SES affected the incidence and RSRs of RCC, which may be beneficial for both better designed clinical trials and efficient prevention methods.

## 1. Introduction

Renal cell carcinoma (RCC) is the most common type of kidney cancer in adults, accounting for approximately 90% of all cases. RCC is classified into three major subtypes including clear cell RCC (ccRCC), papillary RCC (pRCC), and chromophobe RCC (chRCC). Clear cell RCC (ccRCC) is the most common subtype, accounting for around 75% of all cases. Papillary RCC (pRCC) and chromophobe RCC (chRCC) account for 15% and 5%, respectively [1]. CpG island methylator phenotype-associated RCC tumor and metabolically divergent chRCC tumor, as two new subsets, have been found and estimated to be associated with very pessimistic prognoses [2].

RCC is one of the most aggressive cancers by an estimated ∼400,000 new cases and ∼179,000 deaths worldwide, ranking as the sixth and eighth most common malignancy in men and women in 2020, respectively [3]. It was indicated that males had suffered from a higher incidence and a higher mortality of RCC than females [4]. Besides, younger black patients were shown to have poorer overall survival than whites [5]. However, these researches were more or less limited by the sample size, selected study variables, or research time span, and did not fully analyze or illustrate the risk factors for RCC. In order to carry out further analysis and make comprehensive information, a long-time span of research seems crucial to be engaged to generate insights for the prevention and treatment of RCC.

SEER is a population-based and comprehensive database in the United States that includes high-quality information for cancer incidence and patient survival data. It began collecting data on cancer cases since 1973, providing information on patient demographics, primary tumor site, tumor morphology and stage at diagnosis, first course of treatment, and follow-up for vital status [6]. Therefore, we collected RCC patients' data from the SEER database to develop the research of risk factors for RCC based on a long period from 1977 to 2016.

## 2. Materials and Methods

### 2.1. Data Source and Patient Selection

All data in this study was obtained from the Surveillance, Epidemiology, and End Results (SEER) database (available at https://seer.cancer.gov/). The data of incidence and survival of RCC patients were collected from nine original SEER sites. Cases diagnosed with RCC between 1977 and 2016 were enrolled, which were selected by the variables of: primary site code: C64.9—Kidney, NOS; ICD-O-3 histologic codes: 8310 and 8312 for Clear Cell, 8260 for papillary and 8317 for chromophobe [7, 8]; sex (male and female); race (blacks, whites, and Others: American Indian/AK Native, Asian/Pacific Islander); Age at diagnosis (0–29, 30–39, 40–49, 50–59 and 60+); and SES (low poverty, medium poverty, and high poverty). Area SES was determined by the county poverty rate [9] which was defined as follows: areas with low poverty (less than 10% of the population below the poverty level); areas with medium poverty (10 to 19.99% of the population below the poverty level); and areas with high poverty (20% or more of the population below the poverty level). Considering that patients in high poverty group were few, we integrated the medium– and high-poverty into one group. All cases in this study were primary and the first diagnosed with RCC. Cases diagnosed by autopsy and died from RCC reported in death certificates were not included.

### 2.2. Statistical Analysis

Summary statistics included incidence and relative survival rates of patients diagnosed with RCC from 1977 to 2016. Because data of the 120-month RSRs in the last ten years was incomplete, the RSRs in the fourth decade were estimated using the polynomial method according to data derived from 2007 to 2015. For the calculation of the incidence and survival rate, patients whose poverty rate by country or race was accordingly defined as “unknown” or “blank” were excluded. Kaplan–Meier curves were conducted to show survival differences among subgroups, which were assessed using a two-tailed log-rank test. Cox proportional hazards models were conducted respectively to evaluate the effect of gender, age, race, and SES on overall survival. A two-tailed *p* < 0.05 was considered statistically significant. GraphPad Prism 8.0.1 software and Stata 12.0 software were used for all data analysis, and a two-tailed *p* value < 0.01 was valuable relating to statistics.

## 3. Results

### 3.1. Trends in Incidence of Renal Cell Carcinoma

**Figure 1:**
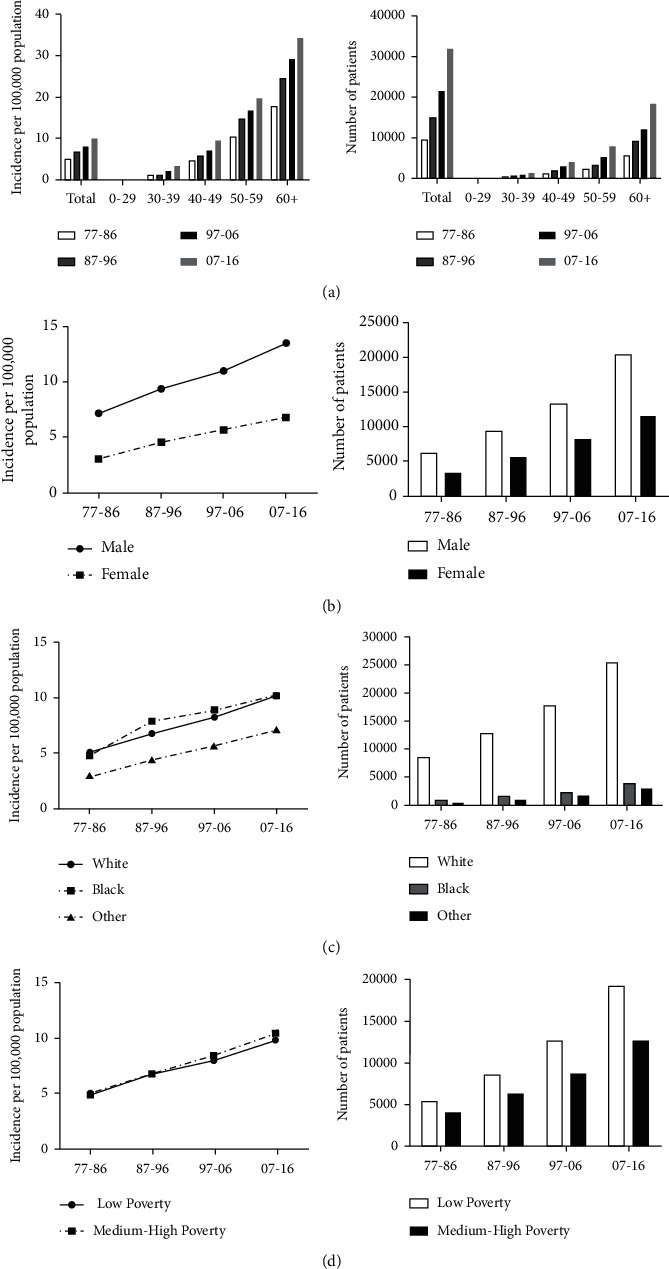
Summary incidence of patients diagnosed with RCC from 1977 to 2016 at the original nine SEER sites. Incidence and number of RCC cases are shown by age groups and decades (a). Incidence and number of RCC cases are grouped by sex (b), race (c), and SES (d), respectively.

To analyze the trend and risk factors of RCC, a total of 77,513 patients diagnosed with RCC between 1977 and 2016 were enrolled in this study. As illustrated, the incidence of RCC remained increasing during the period of 1977–2016 (4.9, 6.7, 8.1, and 9.9, Supplementary Table S1). It increased remarkably in patients over 60 years old (17.6, 24.4, 29.2, and 34.4, Supplementary Table S1), which was the highest in all age groups (Supplementary Table S1 and Figure 1(a)). Moreover, incidence in male group was substantially higher than that in female group (7.2 vs. 3.1 in 1977–1986, 9.4 vs. 4.6 in 1987–1996, 11.0 vs. 5.7 in 1997–2006 and 13.5 vs. 6.8 in 2007–2016; Supplementary Table S1 and Figure 1(b)). Incidence in all race groups also exhibited an increasing trend (5.1, 6.8, 8.3, and 10.2 in whites; 4.8, 7.9, 8.9, and 10.2 in blacks; Supplementary Table S1 and Figure 1(c)). Compared with whites and blacks, the incidence in other groups which included American Indian/AK Native and Asian/Pacific Islander was rather lower (3.0, 4.4, 5.7, and 7.2; Supplementary Table S1 and Figure 1(c)). When analyzed based on socioeconomic status, all the groups showed an increased incidence, and the incidence in the low-poverty group was slightly higher than that in the medium-high-poverty group (Figure 1(d)). Together, these data suggested a stable increase of incidence of RCC in the past decades and that older people, males, black people, and poorer people had a higher incidence of RCC.

### 3.2. RSRs of Renal Cell Carcinoma

**Figure 2:**
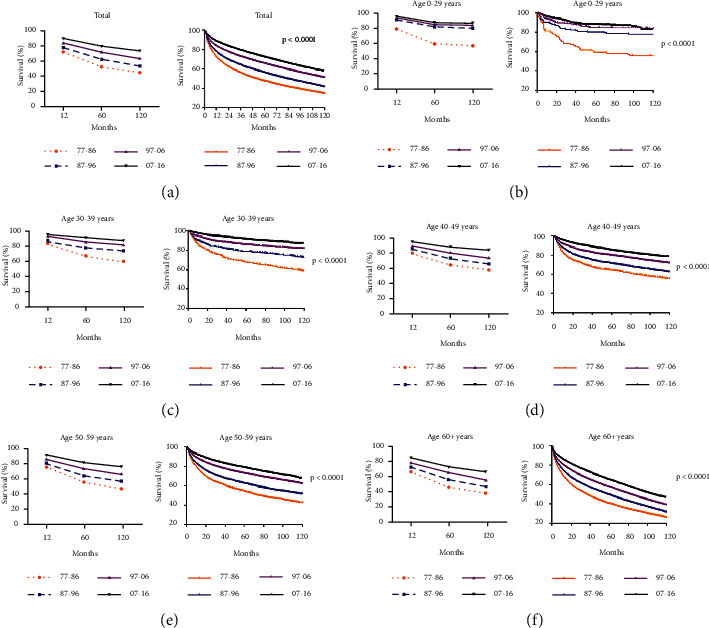
Trends in RSRs (a) (b), (c) (d), and (e) (f) and Kaplan–Meier survival analysis (a) (b), (c) (d), and (e) (f) for patients with RCC from 1977 to 2016 at eighteen SEER sites according to age groups.

Next, we investigated the change of RSRs of patients with RCC. We found that one-year survival, five-year survival, and ten-year survival of patients with RCC had increased stably between 1977 and 2016, and the uptrend could be found in all age groups (Figure 2(a), 2(b)–2(f)). Among these groups, patients older than 60 years had the lowest survival rate (39.8, 48.3, 57.3, and 67.6 for 120-month RSRs; Supplementary Table S2 and Figure 2(f), (f)). The median survival for these patients was 36 months in 1977–1986, 58 months in 1987–1996, 82 months in 1997–2006, and 105 months in 2007–2016 (Figure 2(f)).

### 3.3. Survival Analysis of RCC Based on Sex

**Figure 3:**
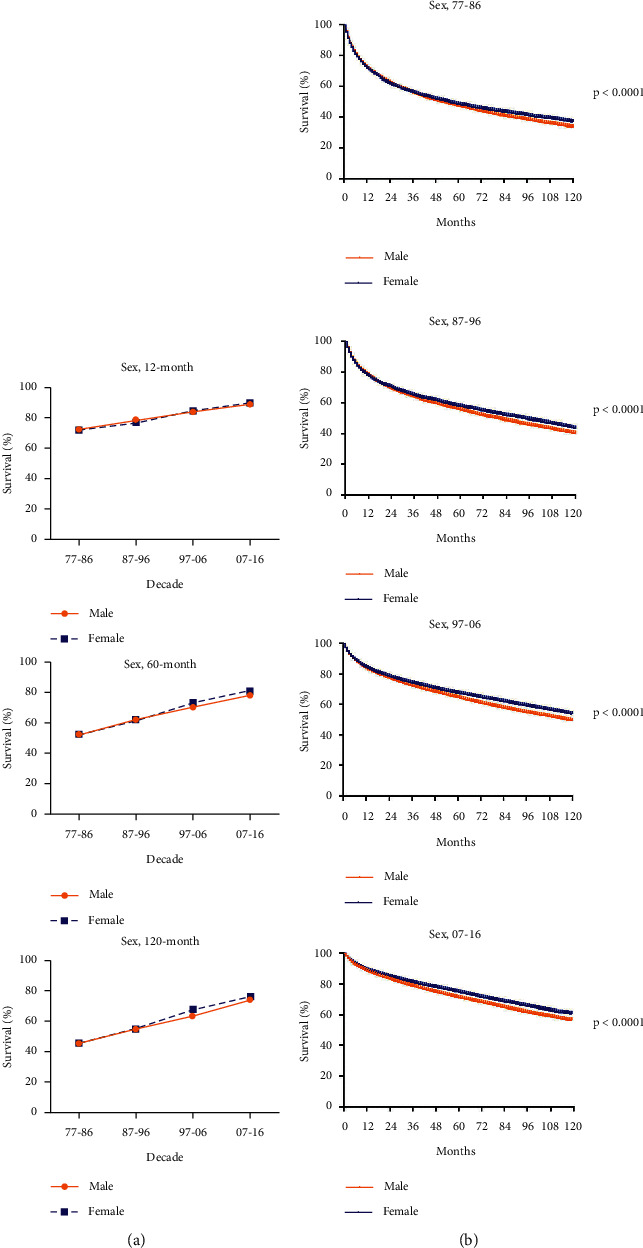
Trends in 12-, 60-, and 120-month RSRs (a) and Kaplan–Meier survival analysis (b) for patients with RCC from 1977 to 2016 at eighteen SEER sites in sex groups.

**Figure 4:**
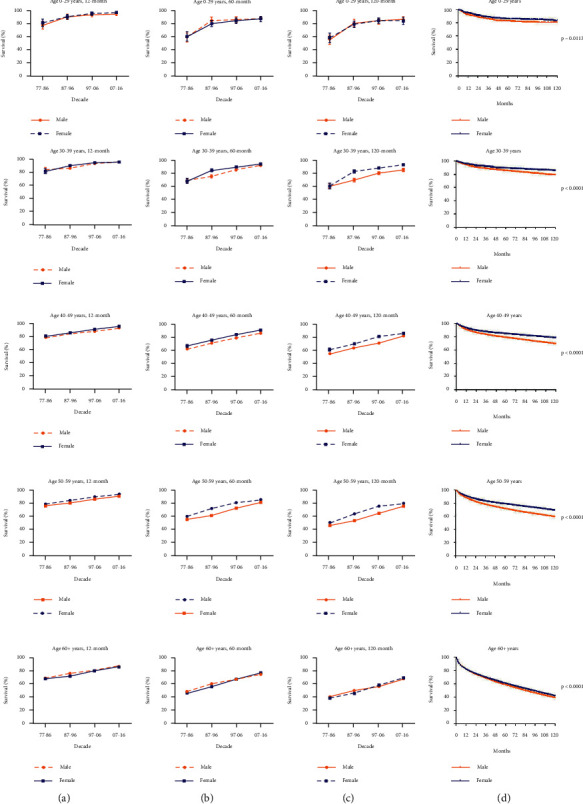
Trends of sexual disparities in 12- (a), 60- (b), 120-months RSRs (c), and Kaplan–Meier survival analysis of sexual disparities (d) for patients with RCC from 1977 to 2016 at eighteen SEER sites according to age groups.

We further investigated the impact of sex on RSRs. It was showed that RSRs in 12, 60, and 120 months had raised in both male and female patients (Figure 3(a)). And, RSRs in female were significantly higher than that in male in every decade (*p* < 0.0001; Figure 3(b)). When analyzed by age groups according to sex, we could find that the survival disparity between male and female was particularly obvious among patients who were 30–59 years old, while not among those younger than 29 years or older than 60 years (Figures 4(a), 4(b), and 4(c). For patients aged 30–59 years, the survival disparity between genders showed to be time-dependent. As the survival time increased, from 12 months to 60 months to 120 months, RSRs in female aged 50–59 years were getting more obviously higher than that in male (from 3.0 to 4.9 to 5.4 higher in 1977–1986, from 3.7 to 11 to 10.4 higher in 1987–1996, from 3.3 to 8.2 to 10.7 higher in 1997–2006, and from 2.6 to 4.7 to 3.7 higher in 2007–2016; Figure 4(a), 4(b), 4(c) and Supplementary Table S3). This trend could also be seen in 30–39 and 40–49 age groups. Similar observations were obtained by using Kaplan–Meier survival analysis. The survival gap between male and female was getting wider over time in the 30–39, 40–49, and 50–59 age groups (*p* < 0.0001; Figure 4(d)). For 0–29 and 60+ age groups, the difference was subtle though there was a survival advantage for female (*p*=0.0113 in 0–29 age group, *p* < 0.0001 in the 60+ age group; Figure 4(d)). Together, these data demonstrated that RSRs of RCC were higher in female patients and that this disparity was age-related.

### 3.4. Survival Analysis of RCC Based on Race and SES

**Figure 5:**
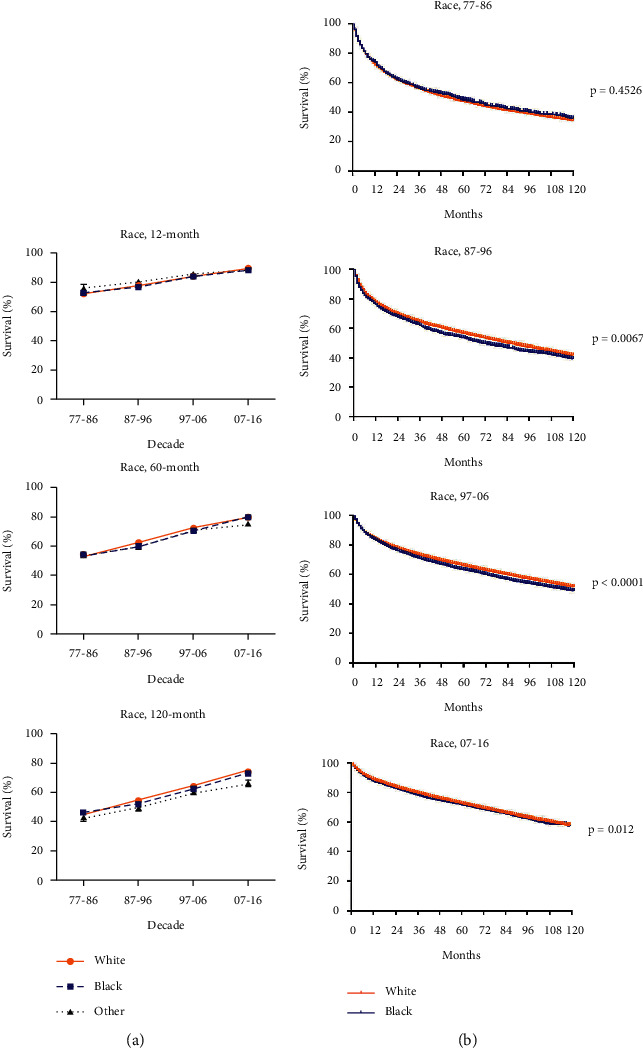
Trends in 12-, 60-, and 120-month RSRs (a) and Kaplan–Meier survival analysis (b) for patients with RCC from 1977 to 2016 at eighteen SEER sites in race groups.

To analyze the impact of race and SES on the RSRs of patients with RCC, we then grouped all the patients into three race groups and two SES groups. Over periods, RSRs in 12, 60, and 120 months in whites, blacks, and Others (American Indian/AK Native and Asian/Pacific Islander) groups increased (Figure 5(a)). In 1977–1986, RSRs in the other group decreased from the highest to the lowest as the survival time expanded (76.4 in 12 months, 53.8 in 60 months, and 43.0 in 120 months; Figure 5(a) and Supplementary Table S4). This change lasted in the next two decades, 1987–1996 and 1997–2006. However, in 2007–2016, RSRs in the other group remained the lowest (88.1 in 12 months, 75.3 in 60 months, and 66.0 in 120 months; Figure 5(a) and Supplementary Table S4). RSRs in whites were higher than that in blacks (*p*=0.0067 in 1987–1996, *p* < 0.0001 in 1997–2006, *p*=0.012 in 2007–2016; Figure 5(b)), except the 1977–1986 group (*p*=0.4526; Figure 5(b)). Unlike disparities in different sex groups, the race disparity between whites and blacks was not affected by the survival time (Figure 5(a), 5(b)). Besides, the survival gap between the two groups narrowed down in 2007–2016 (Figure 5(b)).

**Figure 6:**
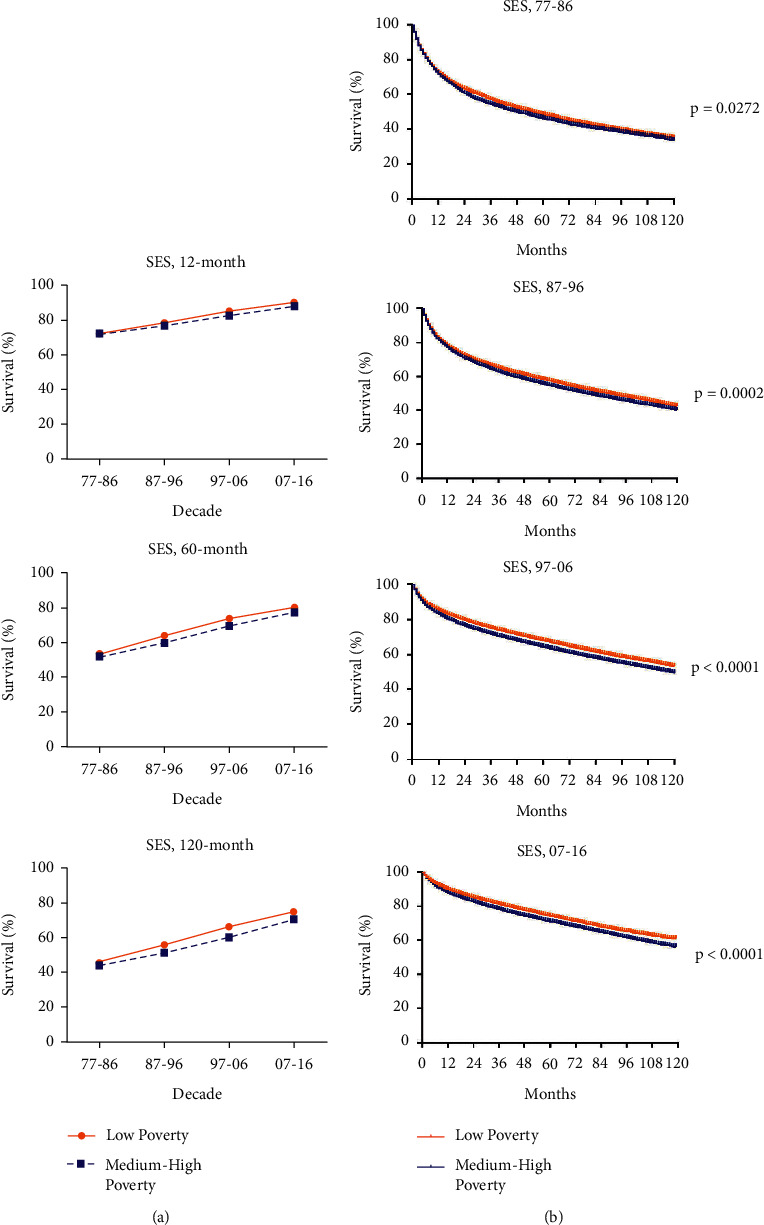
Trends in 12-, 60-, and 120-month RSRs (a) and Kaplan–Meier survival analysis (b) for patients with RCC from 1977 to 2016 at eighteen SEER sites in SES groups.

When grouped by SES, RSRs of patients with RCC showed an increasing trend during the long period (Figure 6(a)). RSRs in low-poverty group were higher than the other group and this disparity was getting more obvious as the survival time increased (*p* = 0.0011, 1987–1996; *p* < 0.0001, 1997–2006; *p* < 0.0001, 2007–2016; Figure 6(b)). And also, the gap between two SES groups was getting wider over the four decades. The median survival in the medium-high poverty group and low poverty group was 48 versus 55 months in 1977–1986, 78 versus 89 months in 1987–1996 and 118 versus 136 months in 1997–2006 (Figure 6(b)). It was worth noting that the interracial distribution of the SES groups was different and there were more whites than blacks classified as low poverty individuals and more blacks than whites as medium-high poverty individuals (Supplementary Figure S1). Together, all data demonstrated that black patients and poorer patients suffered from lower RSRs of RCC.

To assess whether these factors we mentioned above exerted an independent impact on RSRs of RCC, we conducted a Cox regression analysis. It revealed that sex, age, and SES were independent factors affecting the survival of RCC in each decade (*p* < 0.05; Table 1). Race was suggested to be an independent factor in the latter three decades (*p* < 0.001 in 1987–1996, *p*=0.001 in 1997–2006, *p* < 0.001 in 2007–2016; Table 1). Hazard ratio suggested that male, older patients, black patients, and medium-high poverty patients were related with shorter survival duration (Table 1).

## 4. Discussion

During the past four decades from 1977 to 2016, the incidence of renal cell carcinoma has increased steadily, and so has RSRs. In addition, it was suggested that males, blacks, and people with medium-high poverty had a higher risk of incidence and lower RSRs from RCC. This survival disparity between sex groups was age-related.

In this study, we found that the incidence of patients with RCC had doubled in the last forty years, from 4.9 to 9.9. This might be explained by the increasing risk factors related to RCC development. Researches have revealed that genetic predisposition/hereditary disorders, smoking, natural/manmade radioactivity, obesity, and hypertension all play their part in the development of RCC [10–13]. Some of the risk factors have prevailed in the past decades, resulting in people being more vulnerable to RCC attack. For example, obesity was confirmed to be independently associated with RCC (body mass index ≥35 vs < 25 kg/m^2^: HR: 1.71, 95% CI: 1.06–2.79) [14, 15]. The prevalence of obesity continued to spread from 1980 to 2015 [16], consistent with the increase in the incidence of RCC from 1977 to 2016. This information reminds us to change our unhealthy lifestyles and avoid hostile environments in time. Besides, along with the promotion of precise test methods such as CT, MRI, and ultrasound, there were more possibilities for us to discover patients with RCC, especially the small and localized tumors [17, 18], which accounted for part of the increased incidence.

RSRs of RCC had a similar increase in the last forty years, especially the long-time survival, 120-month RSRs, from 44.8 to 73.2. Developed technology and improved medical treatment may play their parts in this process. Nephrectomy is the classic way to treat RCC, which is typically reserved for localized disease [1]. For inoperable or metastatic RCC, ablative therapies and immunotherapies have been applied to clinical treatment and are well developed [19]. These treatments enable patients with RCC to be better cured or have a longer survival time [20–22]. Moreover, as we mentioned before, more small and localized tumors can be diagnosed, and such renal cell carcinoma is usually a low-grade early tumor [23]. Those patients can be treated at an early stage so as to develop less lymph node involvement and prevent distant metastases, which may improve the prognosis [18,24,25].

Our research further found that there was a gender disparity over the last four decades: females had a lower incidence and a higher survival rate than males. It was reported that compared with females, males were attacked by larger tumors and higher pathological grades, along with a higher incidence of regional or metastatic spread [26], which may be the reason for the gender disparity in survival. Contrary to the findings that there is no gender difference in the survival rate of patients over the age of 59 reported in some studies [27,28], our study showed that in patients older than 60 years, there was still a significantly advantageous survival rate in females. Interestingly, among patients aged 30–59 years, this gender disparity was particularly obvious. Previous researches have demonstrated that sex hormones play an important role in RCC development, which may be helpful for explaining this age-related survival disparity between males and females. Estrogen is able to inhibit proliferation, migration, and invasion and induce apoptosis of RCC cells through ER*β* (estrogen receptor *β*) activation [29], suggesting a protective effect on survival, while androgen is able to promote RCC progression through androgen receptor [30]. We postulate that the combined effect of estrogen and androgen amplifies the gender disparity among patients aged 30–59 years, while this effect gets attenuated in younger or older patients because of lower hormone levels. This important finding adds to the growing evidence that estrogen protects against RCC, indicating a new target for RCC treatment.

This research also showed that white people had better survival than black people although there was no previous research which reported in detail that racial difference could affect biological factors. Interestingly, there is research reporting less chance for blacks to get surgery might provide some sort of explanation for the racial disparity [31]. As what we have demonstrated above, the low-poverty individuals consisted of a majority of white patients, which was reversed in the medium-poverty group. And also, there was a close relationship between RSRs and SES: the survival time of patients with low poverty was the highest, while that of patients with high poverty was the lowest. We then assumed that economic factors may play a certain role in the survival of patients with RCC. Since patients in deprived areas are less likely to have improved healthcare and made an early diagnosis of cancers, they usually have worse survivals. Therefore, patients in the medium-high poverty group and black patients with RCC may have a lower chance of cure and a lower survival rate. To eliminate these disparities, it is not only necessary to improve medical and health conditions but also to develop healthcare regulations to fill the gaps among SES groups.

In this study, a large number of samples were collected from the SEER registries including diverse populations of patients over the past forty decades from 1977 to 2016. In addition to a long time span, this study depicted the incidence and survival of RCC based on multiple aspects: sex, race, and socioeconomic status. We should also state that the statistics we incorporated into our study only represented the selected SEER areas, so the results were limited and should be interpreted carefully when being applied to evaluate the situation of patients in other locations. Also, the findings of this study may be biased if there was any under-registration or misclassification of cases from the SEER database, as well as subsequent changes in socioeconomic status.

## 5. Conclusions

Our study demonstrates an increased incidence and RSRs of renal cell carcinoma from 1977 to 2016. Significant disparities between different populations of patients remain a general trend. Males had a higher incidence and lower survival than females. The survival disparity was shown in all age groups, with a more noticeable change in patients aged 30–59 years. Besides, race and SES were found to be independent factors affecting RCC development, with black and medium-high poverty people suffering from higher incidence and lower survival. This study provides comprehensive information about RCC development based on a large population and a long period, which may be helpful for predicting the future trends of RCC and better designing clinical trials to diminish survival imbalance in different populations. Besides, the doubled incidence serves as a timely reminder of efficient prevention methods.

## Figures and Tables

**Table 1:** Summary data for Cox regression analysis of survival in patients with RCC from 1977 to 2016 at eighteen SEER sites. *P* < 0.05 is considered significant.

Variable	HR (95% CI)	*p* value
*All 1977-1986*		
*Univariate*		
Sex	0.911 (0.871–0.953)	< 0.001
Age	1.042 (1.040–1.044)	< 0.001
Race	0.974 (0.900–1.054)	0.522
SES	1.024 (0.984–1.066)	0.069
*Multivariate*		
Sex	0.843 (0.806–0.881)	< 0.001
Age	1.043 (1.041–1.045)	< 0.001
SES	1.049 (1.004–1.096)	0.032

*All 1987-1996*		
*Univariate*		
Sex	0.918 (0.887–0.950)	< 0.001
Age	1.046 (1.044–1.047)	< 0.001
Race	1.087 (1.029–1.147)	0.003
SES	1.076 (1.041–1.111)	<0.001
*Multivariate*		
Sex	0.846 (0.818–0.876)	< 0.001
Age	1.047 (1.045–1.048)	< 0.001
Race	1.225 (1.153–1.302)	< 0.001
SES	1.121 (1.103–1.154)	< 0.001

*All 1997-2006*		
*Univariate*		
Sex	0.899 (0.878–0.921)	< 0.001
Age	1.049 (1.048–1.050)	< 0.001
Race	1.075 (1.037–1.113)	< 0.001
SES	1.128 (1.103–1.154)	< 0.001
*Multivariate*		
Sex	0.809 (0.791–0.828)	< 0.001
Age	1.050 (1.049–1.051)	< 0.001
Race	1.083 (1.034–1.133)	0.001
SES	1.122 (1.096–1.148)	< 0.001

*All 2007-2016*		
*Univariate*		
Sex	0.885 (0.862–0.909)	< 0.001
Age	1.048 (1.047–1.049)	< 0.001
Race	1.050 (1.010–1.090)	0.012
SES	1.169 (1.139–1.199)	< 0.001
*Multivariate*		
Sex	0.804 (0.782–0.826)	< 0.001
Age	1.049 (1.048–1.050)	< 0.001
Race	1.159 (1.116–1.205)	<0.001
SES	1.175 (1.144–1.206)	< 0.001

## Data Availability

All data, models, and code generated or used during the study appear in the submitted article.

## Abbreviations

CcRCC: Clear renal cell carcinoma
chRCC: Chromophobe renal cell carcinoma
pRCC: Papillary renal cell carcinoma
RCC: Renal cell carcinoma
RSRs: Relative survival rates
SES: Socioeconomic status
SEER: Surveillance, Epidemiology, and End Results
95% CI: 95% confidence interval
HR: Hazard ratio.

## Supplementary Materials

Supplementary Materials Supplementary Figure 1: changes of the distribution of SES in different races across four decades. Supplementary Table 1: the incidence of RCC has been listed according to age group and decade within sex, SES, and race groups from 1977 to 2016 at nine SEER sites. Data is incidence per 100,000 people by year of diagnosis, with the number of patients in parentheses. Supplementary Table 2: relative survival rates of patients with RCC during the periods of 1977–1986, 1987–1996, 1997–2006, and 2007–2016 at nine SEER sites. The following statistics sheet calculates mean ± SEM used to describe the variability within the sample, with number of patients in parentheses. Supplementary Table 3: relative survival rates of RCC patients according to sex, age group, and calendar years from 1977 to 2016 at nine SEER sites. The following statistics sheet calculates mean ± SEM used to describe the variability within the sample, with number of patients in parentheses. Supplementary Table 4: relative survival rates of RCC patients according to race, age group, and calendar period from 1977 to 2016 at nine SEER sites. The following statistics sheet calculates mean ± SEM used to describe the variability within the sample, with number of patients in parentheses.
