# Supplementary figures and images for: Survival Improvement in Patients with Renal Cell Carcinoma and Disparities between Different Sexes, Races, and Socioeconomic Status: 1977–2016
Source: J Oncol. 2022 Jul 30;2022:1587365. doi: 10.1155/2022/1587365 (PMC9356869; doi:10.1155/2022/1587365)

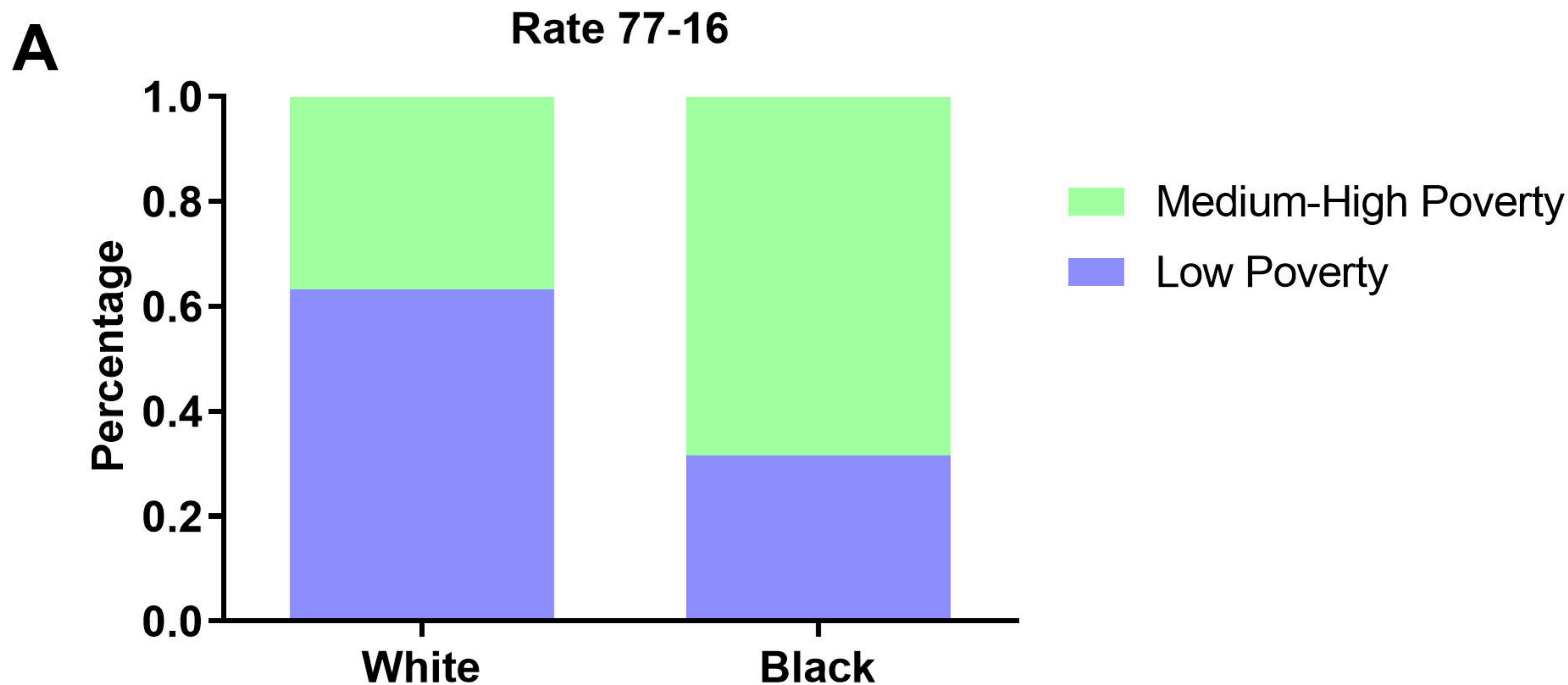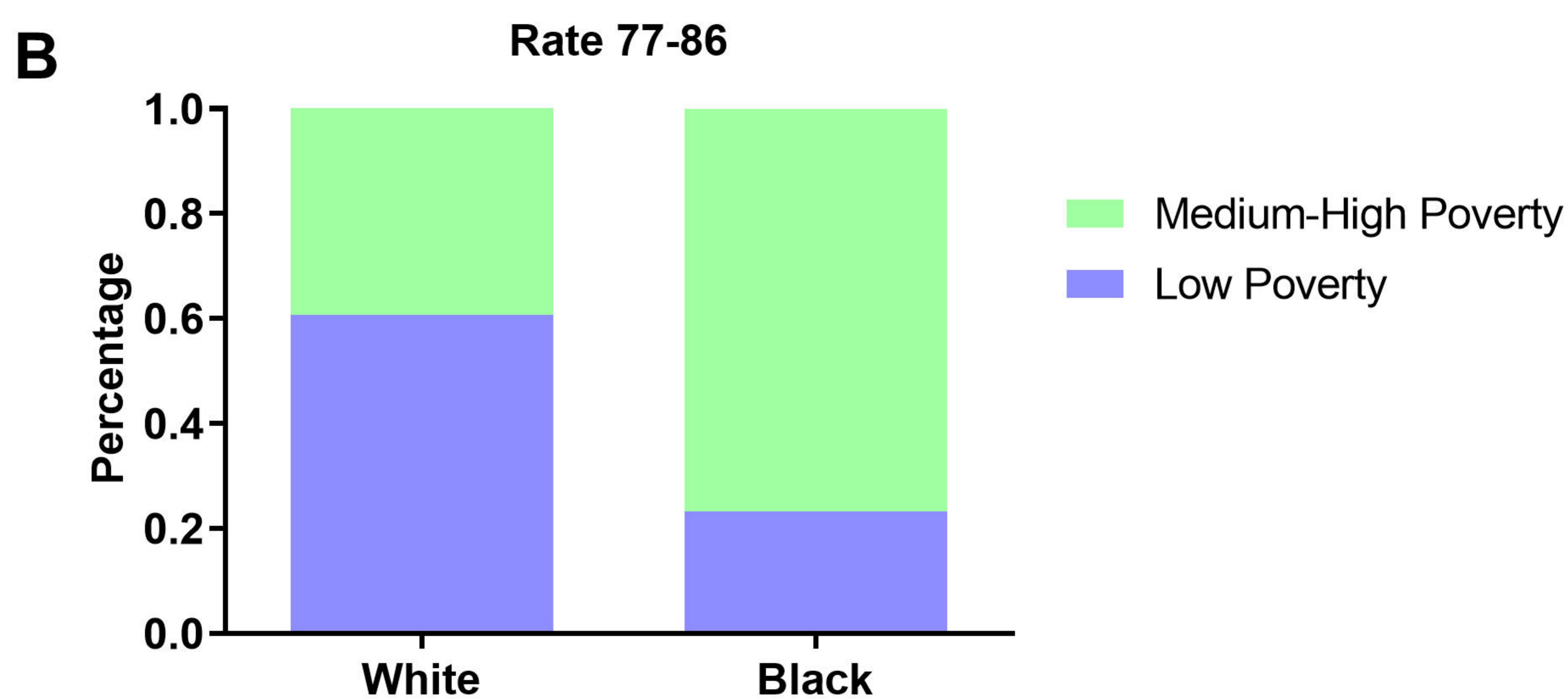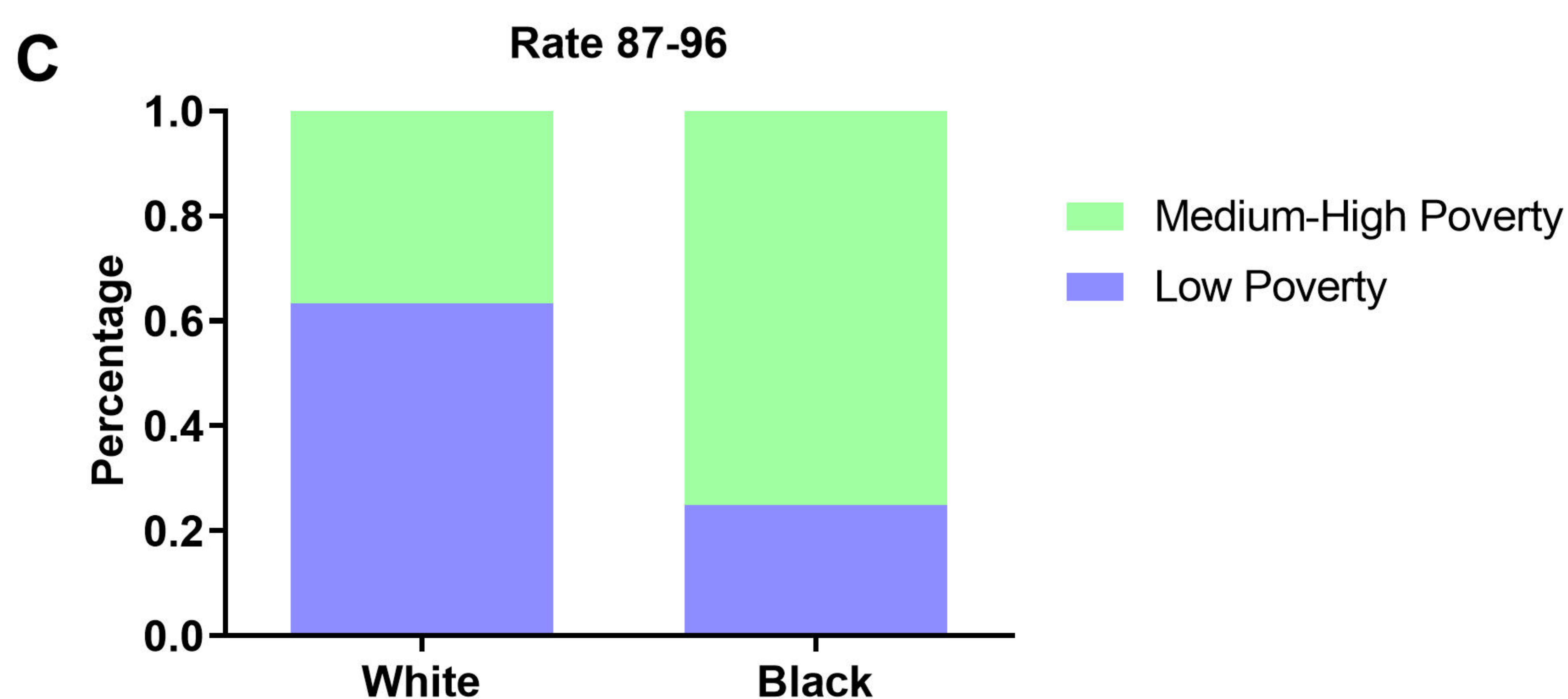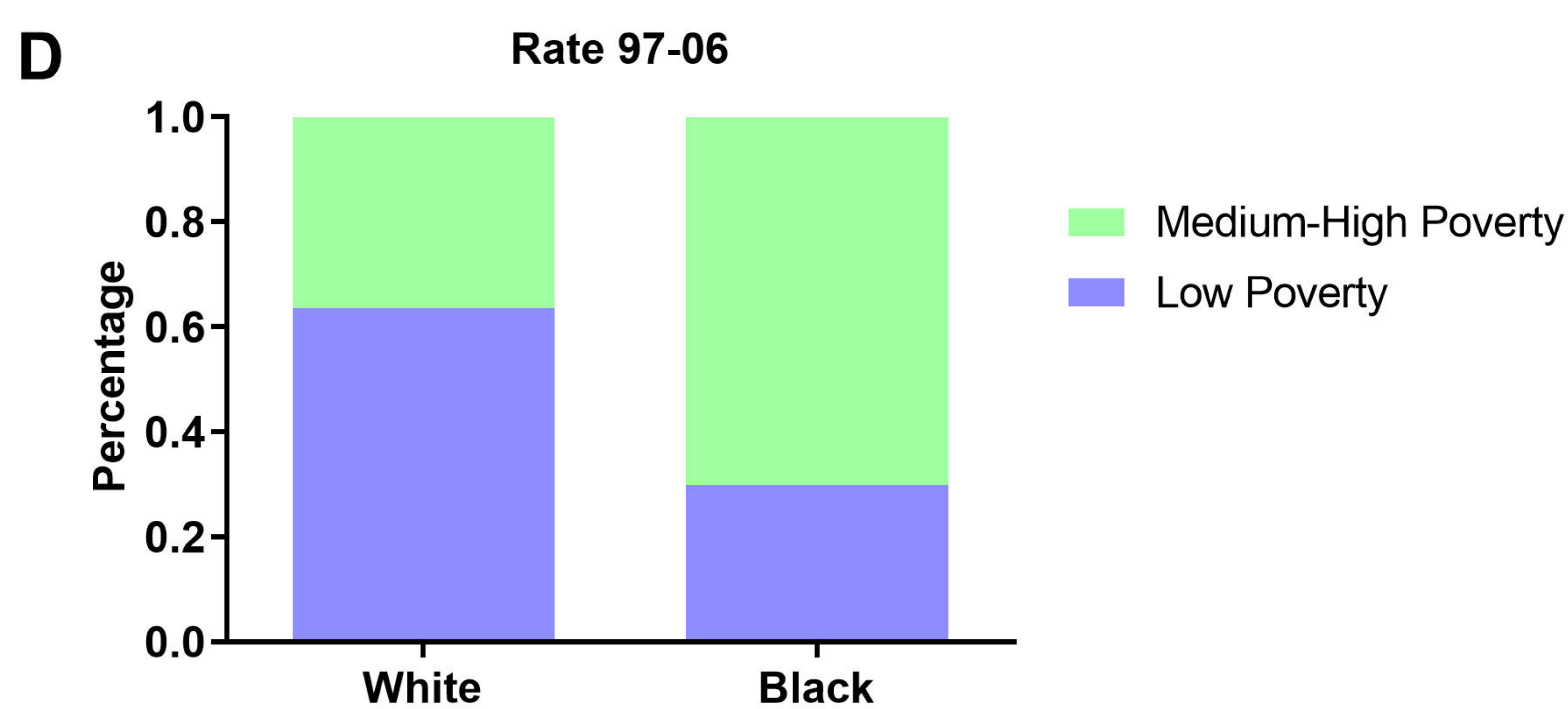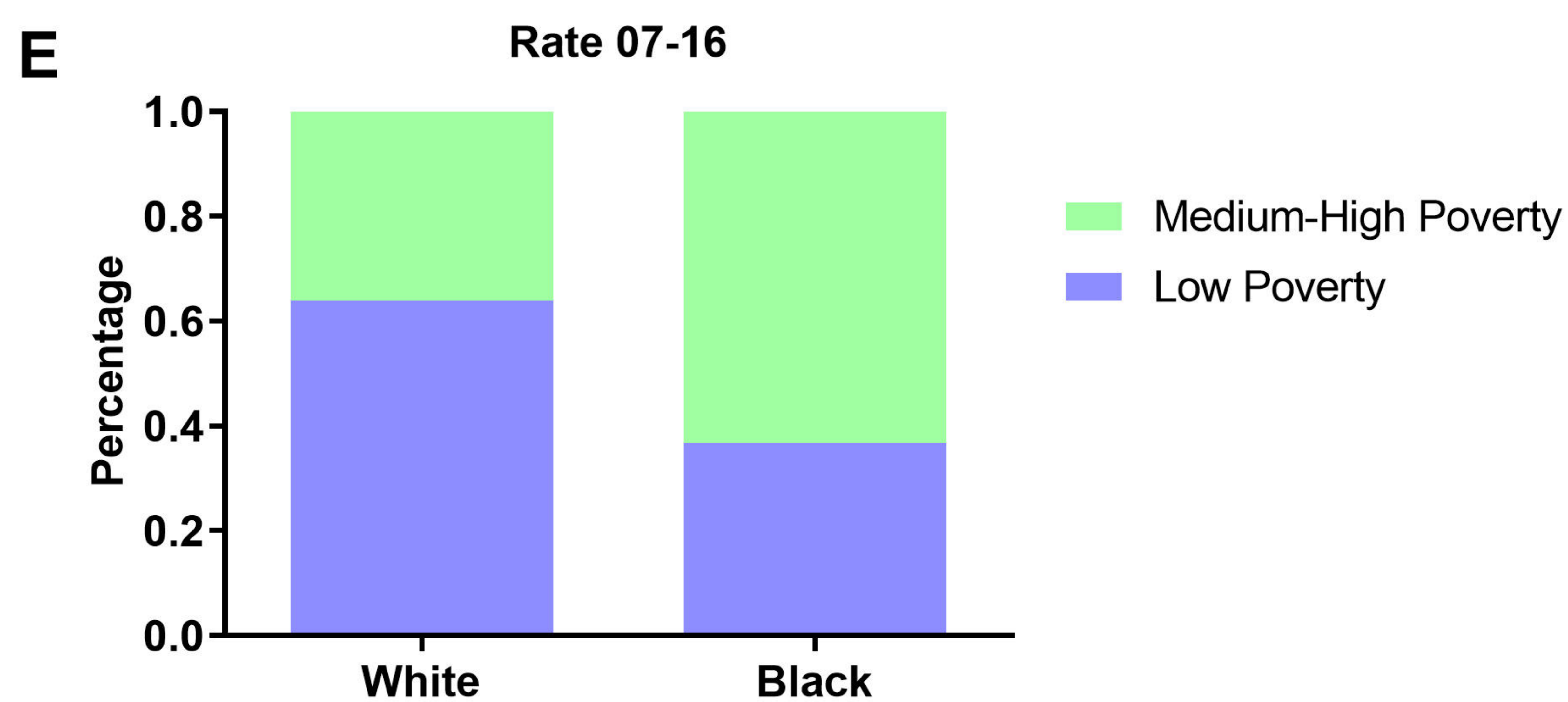

Supplement: Supplementary Materials — Supplementary Figure 1: changes of the distribution of SES in different races across four decades. Supplementary Table 1: the incidence of RCC has been listed according to age group and decade within sex, SES, and race groups from 1977 to 2016 at nine SEER sites. Data is incidence per 100,000 people by year of diagnosis, with the number of patients in parentheses. Supplementary Table 2: relative survival rates of patients with RCC during the periods of 1977–1986, 1987–1996, 1997–2006, and 2007–2016 at nine SEER sites. The following statistics sheet calculates mean ± SEM used to describe the variability within the sample, with number of patients in parentheses. Supplementary Table 3: relative survival rates of RCC patients according to sex, age group, and calendar years from 1977 to 2016 at nine SEER sites. The following statistics sheet calculates mean ± SEM used to describe the variability within the sample, with number of patients in parentheses. Supplementary Table 4: relative survival rates of RCC patients according to race, age group, and calendar period from 1977 to 2016 at nine SEER sites. The following statistics sheet calculates mean ± SEM used to describe the variability within the sample, with number of patients in parentheses. [file 1587365.f1.zip › 1587365.f1/Supplementary Figure 1.pdf]
